# Rethinking the Role of the Public Health Clinic: Comparison of Outpatient Utilization in the Public Health Clinics and Private Clinics in Korea

**DOI:** 10.3390/ijerph15071312

**Published:** 2018-06-22

**Authors:** Agnus M. Kim, Seongcheol Cho, Hyun Joo Kim, Min-Woo Jo, Sang Jun Eun, Jin Yong Lee

**Affiliations:** 1 Department of Health Policy and Management, Seoul National University College of Medicine, Seoul 03080, Korea; agnus@snu.ac.kr; 2 Regional Emergency Care Center, Internal Medicine, Seoul National University Hospital, Seoul National University College of Medicine, Seoul 03080, Korea; mdchosc@gmail.com; 3 Department of Nursing Science, Shinsung University, Dangjin 31801, Korea; hyjkim2012@gmail.com; 4 Department of Preventive Medicine, University of Ulsan College of Medicine, Seoul 05505, Korea; mdjominwoo@gmail.com; 5 Department of Preventive Medicine, Chungnam National University College of Medicine, Daejeon 35015, Korea; 6 Public Health Medical Service, Boramae Medical Center, Seoul National University College of Medicine, Seoul 07061, Korea

**Keywords:** Primary Care, Outpatients, Ownership, Insurance, Coinsurance, Copayment, Public Health Clinic, Korea

## Abstract

Korea has experienced an overall expansion of access to care in the past few decades, which necessitated the reconsideration of the role of the public health clinics (PHC) as a primary care provider. The recent controversy about the outpatient copayment waiver for the elderly in the PHC is in the same vein. This study compared the outpatient utilization of the PHC and private clinics, and investigated its factors. Data were acquired from the National Patient Sample in 2013. Compared with private clinics, the patients in the PHC were more elderly and had less severe conditions. Being elderly, the status of National Health Insurance (NHI) beneficiaries, less comorbidities, and low total claim costs were found to be factors for choosing the PHC over private clinics. These results suggest that the elderly, who are the main beneficiaries of copayment waivers in the PHC, are the most likely to use the outpatient service by the PHC. The functions of the PHC need to be rearranged according to the recent advancements in the health care system in Korea. Diverting the resources and efforts from outpatient care to functions that best serve the health of the population should be considered.

## 1. Introduction

The health care system in Korea has experienced remarkable development in the past decades. The national health insurance achieved universal health coverage only 12 years after its inception in 1977 [1], and the number of health care facilities and hospital beds increased by about 400% and 800% each during the recent three decades [2,3]. This change brought an overall expansion of access to care across the nation [4]. The improvement necessitated the reconsideration of the role of public health facilities, which were believed to care for the underserved population, as the increase in health care facilities and the expanded coverage of national health insurance reduced the need for such roles. The recent controversy about the role of the public health clinics (PHC) in Korea is related to those changes in the health care system in Korea. 

The public health clinic is a community-oriented health care center operated by a local government [5], whose primary role is health promotion and disease prevention for local residents [6]. Established for the purpose of providing medical relief to the poor and controlling infectious diseases in 1946 under the United States Military Government, the PHC have played a vital role in public health concerning sanitation, immunization, family planning, and infectious disease control for the latter half of the 20th century [7]. Providing primary care, especially to underserved populations, was also an important role of the PHC due to the shortage of health care facilities until the 1990s. However, the sharp increase in the health care facilities, which was mainly driven by the private sector [8], lessened the need for the provision of primary care by the PHC.

In the 2000s, outpatient care of the PHC emerged as an issue with the controversy about the copayment waiver in the PHC for elderly patients [9]. Discounting and waiving out-of-pocket expense are prohibited by the law—the Medical Service Act—which considers it as an inducement [10]. However, the PHC are legally exempted from that liability with the approval of the head of the local government [10]. Many local governments allow the PHC to waive and reimburse the copayment for the elderly [11]. This has caused contention between the private clinics and PHC. While the local governments and PHC argue that the waiver is a necessary support for the local community, the private clinics claim that the waiver is an unfair practice that is intended to win the favor of local residents, which hurts private health care providers [12,13]. 

As of 2018, there are a total of 3463 PHC in Korea, including 241 PHC in districts, 1,316 branches of PHC in towns, and 1906 public medical centers where nurses practice instead of physicians in remote rural areas, which is about 11% of the total number of private clinics [14]. While the PHC increased by 9% over the past 30 years, the private clinics increased by 260% [2]. The access to care hugely increased both financially and geographically. Then, what is the reason that the PHC retain the function of a primary care clinic? Is a copayment waiver in the PHC justifiable? To find the answers for these questions, the current practice pattern in the PHC needs to be examined to see if it serves the ultimate purpose of their establishment: promoting the health of the population with the least waste of resources.

Despite the controversies concerning primary care services provided by the PHC [15], little is investigated about the primary care use in the PHC in comparison with the private clinics. However, comparing the utilization pattern of the PHC and the private clinics would be the first step in examining the current controversies about the role of PHC as a primary care provider. This study was performed to compare the outpatient utilization in the PHC and private clinics and investigate the factors that affect the use of the PHC in Korea.

## 2. Materials and Methods 

The National Patient Sample (NPS) data for the 2013 period was used in this study (serial number: HIRA-NPS-2013-0084). The NPS is a nationally representative sample dataset of the Health Insurance Review and Assessment Service (HIRA) in Korea, which collects the claim data of 46 million patients across all ages from almost 80,000 health care service providers in Korea [16]. The NPS is extracted by using a stratified randomized sampling method from the HIRA database, which includes the information about patients’ socio-demographic characteristics, diagnosis, treatment, procedures, and prescription drugs. 

First, 15,351,135 outpatient services claim of the patients, who did not have a history of hospital admission in 2013, were extracted. Second, the selected claim cases were linked to the information on the diagnostic codes for each claim. Finally, we extracted 12,530,101 claims. Each claim was designed to represent 33.3 claims in the 2013 NIS database. This sampling weight was applied to all of the statistical analyses in this study.

The differences among the outpatient visits between private clinics and PHC were analyzed according to gender (male, female), age group (0–19, 20–39, 40–64, 65 and over), type of health insurance (National Health Insurance, Medical Aid), regions where clinics were located, visits of patient with simple or minor disease groups (SMDGs) (yes, no), Charlson comorbidity index (CCI) (zero, one, and more) and healthcare costs. Regions were categorized into metropolitan regions and non-metropolitan regions. Metropolitan regions include Seoul, Busan, Incheon, Daegu, Gwangju, Daejeon, and Ulsan, and nine provinces (Gyeonggi, Gangwon, Chungbuk, Chungnam, Jeonbuk, Jeonnam, Gyeongbuk, Gyeongnam, and Jeju) were categorized as non-metropolitan regions. Fifty-two SMDGs were designated by the Ministry of Health and Welfare in Korea [4,17], which recommended that patients with these conditions visit primary care clinics. The CCI score was calculated by summating the weighted scores for the 17 medical conditions [18]. Healthcare costs were presented as costs reimbursed from the National Health Insurance Service in Korea (NHIS costs), out-of-pocket (OOP) costs paid by a beneficiary, and total claim costs, which is the sum of NHIS costs and OOP costs. The costs from medical services, which were not covered by the NHIS, were not included in the analysis due to the unavailability of the data.

In order to identify the difference in outpatient utilization between the PHC and private clinics, χ^2^ tests were performed. A *t-*test was conducted to examine the difference in annual healthcare costs between the PHC and private clinics. To identify the factors causing the difference in outpatient utilization between the PHC and private clinics, a logistic regression analysis was performed with the type of primary care facilities (PHC, private clinics) as a dependent variable, and the age, gender, type of health insurance, region where medical institution was located, visits of patients with SMDGs, CCI, and healthcare costs were independent variables. The analyses were conducted using SAS, version 9.3 (SAS Institute, Inc., Cary, NC, USA) and SPSS 22 (IBM Corporation, Armonk, NY, USA).

## 3. Results

Descriptive statistics of outpatient utilization of PHC and private clinics are presented in Table 1. The total weighted number of outpatient visits was 417,664,423, of which 3% occurred in the PHC, and 97% occurred in private clinics. Compared with private clinics, the proportion of elderly people in the PHC was significantly high. While 24.5% of patients were elderly (65 and over) in private clinics, 63.9% of patients were elderly in the PHC. Concerning the type of health insurance, the proportion of Medical Aid beneficiaries was 5.4% in the PHC and 4.7% in private clinics. Visits for SMDGs constituted 70.8% of patient visits in the PHC, which was lower than the 79.8% in private clinics. The proportion of patients with a high comorbidity score (CCI of 1 and more) was 6.5% in the PHC and 9.8% in private clinics. Total claim costs, NHI costs, and OOP costs of the PHC was 1.8%, 1.8%, and 1.6% respectively, of private clinics. 

According to the logistic regression results (Table 2), the odds of visiting the PHC rather than private clinics were higher in the older age groups, and the odds were 6.04 in the elderly. Concerning the type of health insurance, the NHI beneficiaries were seven times more likely to visit PHC compared with Medical Aid beneficiaries. Furthermore, the residents of non-metropolitan area were four times more likely to visit the PHC than those of the metropolitan area. Regarding comorbidities, patients with less comorbidities were more likely to visit the PHC, and the patients with the diseases that do not belong to the SMDG were slightly more likely to use the PHC. In terms of total claim costs and OOP costs, the lowest bracket showed a strong tendency to choose the PHC over private clinics for outpatient visits, and this tendency was more prominent in the case of total claim costs. 

## 4. Discussion

In order to examine the role of the PHC as a primary care provider, we compared the outpatient utilization in the PHC and private clinics, and performed a logistic regression analysis with outpatient visits classified by the type of primary care facilities as a dependent variable. According to the study results, being elderly, NHI status, and low total claim costs were found to be strong factors for using the PHC, and people with less comorbidities showed a tendency to choose the PHC over private clinics.

**Figure A1:**
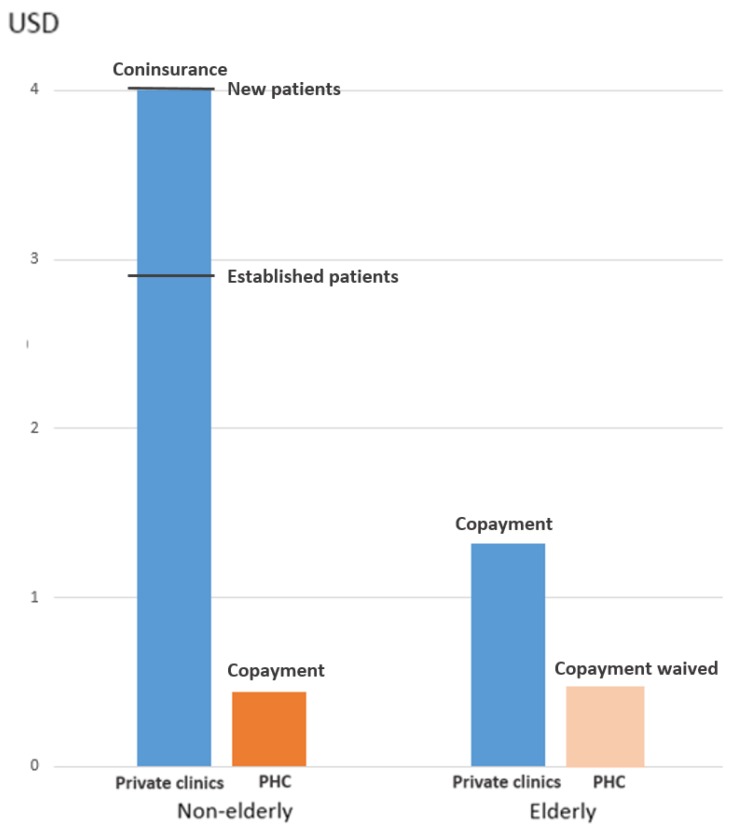
Out-of-pocket costs per outpatient visit in the public health clinics and private clinics.

**Figure A2:**
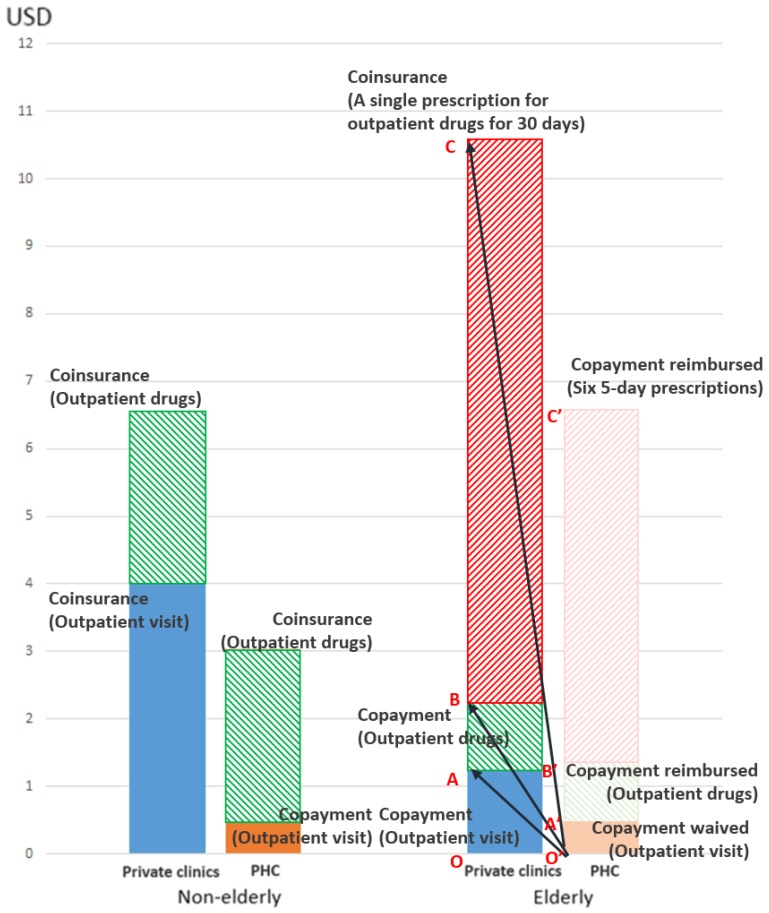
Out-of-pocket expense for an outpatient visit and outpatient drugs in the public health clinics and private clinics. This estimate is when the total cost (OOP + NHI reimbursement) is assumed to be $13 USD, and the fee for drugs is assumed to be $9 USD. PHC: public health clinic, OA: The OOP expense for an outpatient visit saved by visiting a PHC rather than a private clinic, AB: The OOP expense for outpatient drugs saved by visiting a PHC rather than a private clinic, BC: The OOP expense for outpatient drugs prescribed for 30 days, which is saved by visiting a PHC rather than a private clinic (in the case of the most commonly prescribed anti-hypertensive drug in Korea in 2017) [39,40], OA’: The waived OOP expense for outpatient visits in the PHC, A’B’: The OOP expense for outpatient drugs, which is later reimbursed to patients or pharmacies, B’C’: The OOP expense for outpatient drugs for 30 days (Six 5-day prescriptions), which is later reimbursed to patients or pharmacies.

The high proportion of elderly people among the outpatient visits in the PHC and the high odds of the elderly people to choose the PHC over private clinics can be explained in three ways. First, the waiver of copayment might have induced the elderly to choose the PHC by reducing the burden of OOP expenses. This assumption corresponds with the effect of the copayment reduction, which has been observed in previous studies [19,20]. Second, the elderly are likely to be more sensitive to the discounting of the OOP costs than the non-elderly. This is especially relevant considering that the gap in the OOP costs for outpatient visits between the PHC and private clinics is larger in the non-elderly than the elderly (Figure A1). For the non-elderly, the difference in OOP costs between the PHC and private clinics might not be so significant as to compensate for the inconvenience in using the PHC, such as longer distance, a less flexible time schedule, and short consultation time. However, for the elderly, who have relatively more time and less money to spare compared with the non-elderly, the gap in OOP costs could be enough to compensate for the inconvenience. Third, the reimbursement for the outpatient drugs costs could have caused the elderly to choose PHC (Figure A2). In particular, the short-term prescription, which is extensively practiced in the PHC [21,22,23,24], is likely to be a very attractive option for the elderly, given the amount of costs that it can save.

In terms of insurance status, the proportion of Medical Aid beneficiaries in the PHC was only slightly lower than those in private clinics. In the regression analysis, NHI beneficiaries were more likely to choose PHC over private clinics than Medical Aid beneficiaries. Medical Aid beneficiaries in Korea should pay smaller copayment amounts (0.9 USD) when visiting private clinics; however, in the PHC, the services are supposed to be provided free of charge for Medical Aid beneficiaries [25]. Given that Medical Aid beneficiaries can be provided free services in the PHC, the results of this study suggest that the effect of providing free services for Medical Aid beneficiaries was not so significant as to make them choose the PHC. In other words, the amount of copayment for Medical Aid beneficiaries in private clinics might be considered negligible by Medical Aid beneficiaries compared to the advantages of using private clinics in terms of access or quality of service.

What is interesting is why the copayment waiver, if it were truly the main reason for the elderly to choose the PHC, had seemingly little impact on the Medical Aid beneficiaries. It can be due to the difference of the amount saved. While the Medical Aid beneficiaries could save $0.9 USD when choosing the PHC, the elderly NHI beneficiaries could save at least $1.3 USD, and the saving increases to more than twice considering the reimbursement for the drugs. However, despite this observation, a question still remains. If Medial Aid beneficiaries chose private clinics, despite the free services in the PHC, because of the concern for access or quality of care, why did the elderly NHI not? The reason might not be only the difference in the amount of cost saved, but also the characteristics of the services that the elderly receive in the PHC. The following explanation about the difference in the patients’ comorbidities between the PHC and private clinics provides a clue for this. 

Patients with lower CCI were more likely to choose the PHC over private clinics. This seems reasonable, considering the characteristics of private clinics in Korea. The proportion of specialists among physicians practicing in private clinics is over 90% in Korea [26]. As the private clinics could cover a broader range of patients than the PHC in terms of the severity of conditions, the proportion of patients with a lower CCI would be higher in the PHC than in private clinics. However, this result could also be related to the strong tendency of the elderly NHI beneficiaries to choose the PHC over private clinics. With the financial benefit from copayment waiver, the elderly NHI beneficiaries with less comorbidities could choose the PHC, even while sacrificing the quality of care which would be a definite concern in severe cases. 

Finally, concerning total claim costs and low OOP costs, the lower these costs were, the higher the likelihood to visit the PHC. This is related to the factors discussed above. As the elderly NHI beneficiaries with less comorbidities, whose fees are usually contained in the range within which copayment is applied, are the main patients in the PHC, both claim costs and OOP costs are contained in the lowest range. As the high proportion of visits with low total costs and OOP costs in the PHC is related to the copayment waiver, the result is suggestive of the effect of copayment reduction on increasing total health care costs, which has been found in earlier studies [27,28,29]. The impact of the copayment waiver on the total health care costs needs to be investigated further, as an increase in the total health care costs is likely to suggest the leakage of the NHI fund, whose liability for the health care costs remains, regardless of the exemption of the copayment.

Our study results indicate that the current practices of the PHC require a careful examination. First, what is the ground that the waiving of OOP costs in the PHC would not induce patients’ unnecessary utilization, if the private clinics would do? The discounting of OOP costs has been strictly prohibited for private practitioners by law. A recent decision of the Constitutional Court reconfirmed that the Act, which declared the behaviors as inducements, was not against the Constitution [30]. Our study results, showing the high likelihood that the elderly were induced to visit the PHC, suggest that PHC cannot be an exception to the law. Second, who is the beneficiary of the short-term prescription? Despite repeating complaints from the local medical societies [11,12,31], the government decided that the practices could not be considered illegal [32]. However, the short-term prescription is an undesirable practice for both the insured and insurer, as it incurs additional costs to both parties. The short-term prescription of the PHC gives more of a burden to the NHI in proportion to the added frequency than does usual long-term prescription. Furthermore, the waived copayment is not waived, in view of the wealth of the nation, as it is gathered from the local governments and given to the PHC, in an amount that increases proportionally to the increased frequency. Moreover, considering the possible overuse that can be aroused from the copayment waiver, short-term prescription may be even harmful to its beneficiaries. The true beneficiary of short-term prescription is the PHC, which would enjoy the fruit of the increased costs. 

The study results strongly suggest that the current role of the PHC as a primary care clinic needs serious rethinking. Our study showed that the PHC was focusing on the elderly with less serous conditions, and that their current practices indicate that they are likely to exploit the elderly and the NHI. This situation cannot justify their role as a primary care provider, not only because of the high possibility of its replacement by other providers, but also because of the possible harm that it can cause. The resource and efforts hitherto given to outpatient care need to be diverted to more productive services such as preventive care or health promotion. Repositioning their role as a community health center, which focuses on the health promotion of the whole population, is recommended. Concerning the underserved population, supporting them by comprehensive health service rather than outpatient care would be more useful. In order for the PHC to be established as a health center for the community, government support is essential. The central government should redefine the role of PHC by rearranging related laws, and the local governments should put efforts into connecting the PHC into local social services. 

There are some limitations in this study. First, the sub-types of the PHC were not considered in this study. The PHC includes three sub-types of facilities: PHC, Branches of PHC, and public medical centers, which differ in the size and characteristics of their coverage area, including the availability of adjacent private clinics. The utilization pattern can differ, depending on the sub-type of the PHC, which was not reflected in this study. Second, the heterogeneity of private clinics was not considered in this analysis. Although no definite agreement has been reached concerning the issue, it is generally accepted that only several specialties could be considered as primary care providers, the number of which is around half of the total number of private clinics [33,34,35]. As the clinical services provided by the PHC are limited to primary care services, only the private clinics, whose practices are in the realm of primary care could have been more desirable in terms of comparability.

## 5. Conclusions

Our results show that the elderly, who are the main beneficiaries of copayment waiver in the PHC, are most likely to use the outpatient service by the PHC. The current role of the PHC as a primary care clinic needs serious rethinking, and the current practice in the outpatient care of the PHC cannot be justified, as it might eventually harm both the beneficiaries and the payer—the NHI and local governments—by causing unnecessary use. The functions of the PHC need to be rearranged according to the recent advancements in the health care system in Korea. Diverting the resources and efforts from outpatient care to functions that best serve the health of the population should be considered. 

## Figures and Tables

**Table 1:** Characteristics of outpatient visits in the public health clinics and private clinics.

Characteristic	No. of Outpatient Visits (%)			*p*-Value
	Total	PHC ^1^	Private Clinics	
**Total**	417,664,423 (100.0)	11,798,425 (100.0)	405,865,998 (100.0)	
**Age group (years)**				
0–19	77,658,372 (18.6)	232,464 (2.0)	77,425,908 (19.1)	<0.001
20–39	70,478,935 (16.9)	342,464 (2.9)	70,136,471 (17.3)	
40–64	162,393,179 (38.9)	3,685,685 (31.2)	158,707,494 (39.1)	
>64	107,133,938 (25.7)	7,537,812 (63.9)	99,596,126 (24.5)	
**Gender**				
Female	244,815,159 (58.0)	6,900,386 (58.5)	237,914,773 (58.6)	<0.001
Male	172,849,264 (41.4)	4,898,039 (41.5)	167,951,225 (41.4)	
**Type of health insurance**				
NHI ^2^	397,902,947 (95.3)	11,160,933 (94.6)	386,742,014 (95.3)	<0.001
Medical Aid	19,761,477 (4.7)	637,492 (5.4)	19,123,984 (4.7)	
**Region**				
Metropolitan	283,169,896 (67.8)	4,260,084 (36.1)	278,909,813 (68.7)	<0.001
Non-metropolitan	134,494,527 (32.2)	7,538,341 (63.9)	126,956,186 (31.3)	
**Visits for SMDGs ^4^**				
Yes	332,383,732 (79.6)	8,347,599 (70.8)	324,036,133 (79.8)	<0.001
No	85,280,691 (20.4)	3,450,826 (29.2)	81,829,865 (20.2)	
**CCI ^5^**				
0	377,073,942 (90.3)	11,034,668 (93.5)	366,039,274 (90.2)	<0.001
1 and more	40,590,481 (9.7)	763,757 (6.5)	39,826,724 (9.8)	
**Total claim costs** (million USD ^3^) (total (mean ± SD ^6^))	6,827.9 (16.3 ± 67.8)	117.9 (10.0 ± 14.2)	6,710.0 (16.5 ± 68.8)	<0.001
**NHI ^2^ costs** (million USD ^3^) (total (mean ± SD ^6^))	5,161.2 (12.4 ± 60.4)	92.3 (7.8 ± 10.1)	5,069.0 (12.5 ± 61.2)	<0.001
**OOP ^7^ costs** (million USD ^3^) (total (mean ± SD ^6^))	1,666.6 (4.0 ± 8.6)	16.9 (2.2 ± 4.4)	1,641.0 (4.0 ± 8.6)	<0.001

^1^ PHC: Public Health Clinic, ^2^ NHI: National Health Insurance, ^3^ USD: United States dollar, ^4^ SMDG: Small Minor Disease Group, ^5^ CCI: Charlson comorbidity index, ^6^ SD: Standard deviation. ^7^ OOP: Out-of-pocket.

**Table 2:** Factors for choosing the public health clinics over private clinics.

Variable	Adjusted Odds Ratio	95% Confidence Interval (Lower Limit–Upper Limit)	*p*-Value
**Age group (years)**			
0–19	1.00 (Reference)		
20–39	1.71	1.68–1.75	<0.001
40–64	2.82	2.79–2.86	<0.001
>65	6.04	5.97–6.12	<0.001
**Gender**			
Female	1.00 (Reference)		
Male	1.12	1.11–1.12	<0.001
**Type of health insurance**			
Medical Aid	1.00 (Reference)		
NHI ^1^	7.06	6.96–7.15	<0.001
**Region**			
Metropolitan area	1.00 (Reference)		
Non-metropolitan area	4.48	4.48–4.49	<0.001
**CCI ^2^**			
1 or more	1.00 (Reference)		
0	2.84	2.83–2.85	<0.001
**Visits for SMDGs ^3^**			
Yes	1.00 (Reference)		
No	1.22	1.22–1.23	<0.001
**Total claim costs** **(average per visit, USD ^4^)**			
>14.4	1.00 (Reference)		
<9.6	33.31	33.12–33.50	<0.001
9.6–11.6	5.48	5.44–5.51	<0.001
11.6–14.4	0.29	0.28–0.29	<0.001
**Out-of-pocket costs** **(average per visit, USD ^4^)**			
>4.0	1.00 (Reference)		
<1.3	5.97	5.94–6.00	<0.001
1.3–2.9	0.01	0.01–0.01	<0.001
2.9–4.0	0.10	0.099–0.101	<0.001
Insurance ^5^*Age group	1.00 (Reference)		
NHI ^1^*Age1b ^6^	1.46	1.43–1.49	<0.001
NHI ^1^*Age2c ^7^	2.63	2.60–2.67	<0.001
NHI ^1^*Age3d ^8^	7.58	7.47–7.68	<0.001

NHI: National Health Insurance, ^2^ CCI: Charlson comorbidity index, ^3^ SMDG: Small Minor Disease Group, ^4^ USD: United States dollar, ^5^ Insurance: Type of health insurance, ^6^ Age1b: 20–39, ^7^ Age2c: 40–64, ^8^ Age3d: >65.

## Appendix A

We compared the out-of-pocket expense per outpatient visit in the private clinics and PHC on the basis of the fee schedule in 2018. In Korea, which implements National Health Insurance (NHI), cost sharing, mostly in the form of coinsurance, is universally applied [36]. The coinsurance for outpatient visits to private clinics is 30%. For the elderly (65 and over), when the fee is below the threshold ($13 USD), copayment ($1.3 USD) is applied, and when the fee exceeds the threshold, coinsurance (10%, 20%, or 30% according to the amount of the fee) is applied [37]. In the PHC, as long as the outpatient fee is contained within $11 USD, copayment is being applied regardless of age. The usual copayment of outpatient visits in the PHC is $0.4 USD. Figure A1 shows the comparison of the OOP costs per outpatient visit in the private clinics and PHC among the elderly (65 and over) and the non-elderly.

PHC: Public Health Clinic. This estimate is when the total cost (out-of-pocket expense + NHI reimbursement) is assumed to be $13 USD (the threshold over which coinsurance is applied for the elderly).

The difference of OOP costs between the PHC and private clinics among the elderly gets even larger when allowing for outpatient drugs. The OOP costs for outpatient drugs is calculated in the same way, both in the PHC and private clinics. In the case of outpatient drugs for the non-elderly (6–64), coinsurance (30%) is applied. For the elderly (65 and above), if the fee for drugs does not exceed $9 USD, a copayment of $0.9 USD is applied, and when the fee exceeds the threshold, coinsurance (20% or 30% according to the amount of the fee) is applied [38]. Figure A2 shows the OOP costs of the PHC and private clinics for one outpatient visit and outpatient drugs. Supposing that the drugs fee is at $9 USD, the OOP costs for the non-elderly is $2.6 USD (coinsurance rate of 30%), and the OOP costs for the elderly is $0.9 USD (copayment) in both the PHC and private clinics. As the fee for outpatient drugs is paid to the pharmacy, it cannot be directly waived by the PHC. Instead, the PHC can reimburse the elderly or pharmacies for the copayment for drugs. For long-term medications, in order to contain the OOP costs for drugs below the threshold under which copayment is applied, some PHC issue a short-term prescription. For example, in the case of the popular anti-hypertensive drug, 30 days’ prescription normally incurs about $8.50 USD of coinsurance. However, in the PHC, the OOP costs becomes $5.3 USD (a copayment of $0.9 USD multiplied by 6), which is reimbursed by local governments.
